# A Novel Cold-Regulated Cold Shock Domain Containing Protein from Scallop *Chlamys farreri* with Nucleic Acid-Binding Activity

**DOI:** 10.1371/journal.pone.0032012

**Published:** 2012-02-16

**Authors:** Chuanyan Yang, Lingling Wang, Vinu S. Siva, Xiaowei Shi, Qiufen Jiang, Jingjing Wang, Huan Zhang, Linsheng Song

**Affiliations:** 1 The Key laboratory of Experimental Marine Biology, Institute of Oceanology, Chinese 7 Academy of Sciences, Qingdao, China; 2 Graduate School, Chinese Academy of Sciences, Beijing, China; University of South Florida College of Medicine, United States of America

## Abstract

**Background:**

The cold shock domain (CSD) containing proteins (CSDPs) are one group of the evolutionarily conserved nucleic acid-binding proteins widely distributed in bacteria, plants, animals, and involved in various cellular processes, including adaptation to low temperature, cellular growth, nutrient stress and stationary phase.

**Methodology:**

The cDNA of a novel CSDP was cloned from Zhikong scallop *Chlamys farreri* (designated as CfCSP) by expressed sequence tag (EST) analysis and rapid amplification of cDNA ends (RACE) approach. The full length cDNA of CfCSP was of 1735 bp containing a 927 bp open reading frame which encoded an N-terminal CSD with conserved nucleic acids binding motif and a C-terminal domain with four Arg-Gly-Gly (RGG) repeats. The CSD of CfCSP shared high homology with the CSDs from other CSDPs in vertebrate, invertebrate and bacteria. The mRNA transcripts of CfCSP were mainly detected in the tissue of adductor and also marginally detectable in gill, hepatopancreas, hemocytes, kidney, mantle and gonad of healthy scallop. The relative expression level of CfCSP was up-regulated significantly in adductor and hemocytes at 1 h and 24 h respectively after low temperature treatment (*P*<0.05). The recombinant CfCSP protein (rCfCSP) could bind ssDNA and in vitro transcribed mRNA, but it could not bind dsDNA. BX04, a cold sensitive *Escherichia coli* CSP quadruple-deletion mutant, was used to examine the cold adaptation ability of CfCSP. After incubation at 17°C for 120 h, the strain of BX04 containing the vector pINIII showed growth defect and failed to form colonies, while strain containing pINIII-CSPA or pINIII-CfCSP grew vigorously, indicating that CfCSP shared a similar function with *E. coli* CSPs for the cold adaptation.

**Conclusions:**

These results suggest that CfCSP is a novel eukaryotic cold-regulated nucleic acid-binding protein and may function as an RNA chaperone in vivo during the cold adaptation process.

## Introduction

All living organisms must adapt to changes in the environment, such as cold shock. Increasing evidence has confirmed the importance of cold-induced proteins as molecular chaperones involved in the cold adaptation [1]–[3]. Among these proteins, cold shock domain (CSD) containing proteins (CSDPs) are one group of the evolutionarily conserved nucleic acid-binding proteins and they are widely distributed in bacteria, plants, and animals [4]–[6]. These CSDPs are involved in various cellular processes, including adaptation to low temperatures, cellular growth, nutrient stress and stationary phase [7].

In prokaryotes, the members of CSDPs are called cold shock proteins (CSPs) and they have been extensively studied in *Escherichia coli*. These proteins consist of a cold shock domain (CSD) with two consensus RNA-binding motifs (RNP1 and RNP2) [8]–[10] and they are responsible for cold shock response. There are nine CSP genes identified from *E. coli*, and four (CSPA, CSPB, CSPG and CSPI) of them can be induced by cold shock [3]. CSPA, the most predominant CSP, may accumulate up to 10% of total proteins after low temperature exposure [11]. BX04, an *E. coli* strain with quadruple-deletion of CSPA, CSPB, CSPG and CSPE can not grow at low temperature [12], [13]. Under low temperature, bacterial CSPs can bind to RNA and destabilize the secondary structures of RNA to prevent the premature transcription termination. CSPA, CSPC, and CSPE have been confirmed to possess *in vivo* and *in vitro* transcription antitermination activity [14]. The CSPA mRNA is able to sense the temperature downshifts, and adopt functionally distinct structures at different temperature, even without the aid of trans-acting factors [15].

In eukaryotes, the CSDPs display multiple functions with the structural features of variable N-terminal sequences [16], diverse auxiliary C-terminal domains and a highly conserved CSD [7], [17]. Based on the C-terminal domain, eukaryotic CSDPs are divided into three classes. The most extensively studied eukaryotic CSDPs are the Y-box (YB) proteins with C-terminal basic/aromatic islands as transcription factors to regulate gene expression [18]–[25]. For example, both the human YB-1 [19], [20] and the *Xenopus* FRGY2 [21]–[24] function as components of the messenger ribonucleoprotein complex (mRNP) to regulate translation. Another class of eukaryotic CSDPs includes LIN-28 from *Caenorhabditis elegans* [25] and a group of glycine-rich plant proteins [26] with C-terminal retroviral-type zinc fingers. LIN28 are involved in the enhancement of translation [27], biogenesis of miRNA [28] and generation of induced pluripotent stem cells [29]. Most of the plant CSDPs, such as AtCSP2 [30], [31], AtCSP3 [32] and WCSP1 [33], [34], act as RNA chaperones in response to low temperature. The third class of eukaryotic CSDPs are identified in a range of invertebrates [35]–[39] with the typical feature of RGG motif, which is defined as closely spaced Arg-Gly-Gly repeats separated by other, often aromatic amino acids [40]. However, the information about the function of invertebrate CSDPs is very limited. ApY1 from *Aplysia californica* [35] and YPS from *Drosophila melanogaster* [37] are reported to interact with RNA *in vitro*, and RBP16 from *Trypanosoma brucei* is involved in kinetoplastid RNA editing and/or translation [41], [42].

Compared with those in prokaryotes, the study on eukaryotic CSDPs, especially on those of invertebrate is still at the beginning, and there is no report about the involvement of the invertebrate CSDPs in the cold shock response. Zhikong scallop (*Chlamys farreri*), one of the most important cultured scallop species in North China, has developed protective mechanisms in response to the cold stress in winter. In the present paper, a cold shock domain containing protein (CfCSP) was identified from *Chlamys farreri*. The mRNA transcripts of CfCSP in different tissues and its temporal expression in adductor and hemocytes after acute cold shock treatment were investigated. The *in vitro* nuclear acids binding activity of the recombinant protein and the *in vivo* functional complementation of bacterial mutants were also examined to characterize its roles in the cold shock response of scallop.

## Materials and Methods

### Ethics statement

The scallops used in the present study are marine cultured animals, and all the experiments are conducted according to the regulations of local and central government.

### Scallop, cold shock treatment and tissue collection

Adults of scallop *C. farreri* with an average 55 mm of shell length were collected from a farm in Qingdao, Shandong Province, China, and maintained in the aerated seawater at 16°C for a week before processing.

For the tissue distribution analysis of CfCSP mRNA, six tissues, including gill, hepatopancreas, kidney, mantle, gonad and muscle from five healthy adult scallops were collected. Hemolymph from these five scallops was collected from the adductor muscle and then immediately centrifuged at 800×g, 4°C for 10 min to harvest the hemocytes. All these tissue samples were stored at −80°C after addition of 1 mL TRIzol reagent (Invitrogen) for subsequent RNA extraction.

Forty scallops were employed in the acute cold shock treatment experiment. Thirty five scallops were cultivated in 24 L tanks containing aerated seawater at 4°C, and other 5 scallops were still kept in 24 L tanks containing aerated seawater at 16°C as the blank group. Five individuals were randomly collected from the experimental group at 1, 3, 6, 12, 24 and 26 h after they were cultivated at 4°C. Muscle and hemocytes from the scallops were collected and stored as described above.

### RNA isolation and cDNA synthesis

Total RNA was isolated from the tissues of scallops using Trizol reagent (Invitrogen). The first strand cDNA synthesis was carried out based on Promega M-MLV RT Usage information using the DNase I (Promega)-treated total RNA as template and oligo (dT)-adaptor primer P1 (Table 1). The reaction mixtures were performed at 42°C for 1 h, terminated by heating at 95°C for 5 min, and subsequently stored at −20°C.

**Table 1:** Primers used in this study.

Primer	Sequence (5′-3′)
Clone primers	
P1 (oligo (dT)-adaptor)	GGCCACGCGTCGACTAGTACT_17_
P2 (CfCSP primer)	TAGGAAAGAAACACCAACTCACTCG
P3 (T7)	GTAATACGACTCACTATAGGGC
Sequencing primers	
P4 (M13-47)	CGCCAGGGTTTTCCCAGTCACGAC
P5 (RV-M)	GAGCGGATAACAATTTCACACAGG
RT primers	
P6 (CfCSP-RTF)	GACTGCCATTACAAAGAACAACCC
P7 (CfCSP-RTR)	GCCAGGTCCTCCTCGGTAGTAA
P8 (β-actin-RTF)	CAAACAGCAGCCTCCTCGTCAT
P9 (β-actin-RTR)	CTGGGCACCTGAACCTTTCGTT
Transcription primers	
P10 (Luc-mRNAF)	GCGTAATACGACTCACTATAGGATGGAAGACGCCAAAAACAT
P11 (Luc-mRNAR)	TTACACGGCGATCTTTCCGC
Recombination primers	
P12 (CfCSP-ReF1)	TTAGAGCTCGGACAAATGGTAACACCTTTATTA
P13 (CfCSP-ReR1)	AGAAAGCTTCCAGCGAGAGATGCTTACT
P14 (CfCSP-ReF2)	TTACATATGGGACAAATGGTAACACCTTTATTA
P15 (CfCSP-ReR2)	AGAGAATTCCCCAGCGAGAGATGCTTACT
P16 (CSPA-ReF)	TTACATATGATGTCCGGTAAAATGACTGGTATC
P17 (CSPA-ReR)	AGAGAATTCCAGGCTGGTTACGTTACCAGCT

### Cloning of the full-length CfCSP cDNA

BLAST analysis of all the EST sequences from the *C. farreri* cDNA library [43] revealed that one EST (no. rscag0_004862, 1373 bp) was homologous to previously identified CSDPs in other animals and a gene specific sense primer P2 (Table 1) was designed to clone the full sequence cDNA of CfCSP by rapid amplification of cDNA ends (RACE) approach. The 3′ end of CfCSP cDNA was obtained using primers P2 and P3 (Table 1). The PCR product was cloned into the pMD18-T simple vector (TaKaRa) and sequenced with primers P4 and P5 (Table 1). The resulting sequences were verified and subjected to cluster analysis.

### Sequence analysis

The cDNA sequence and deduced amino acid sequence of CfCSP were analyzed using the BLAST algorithm (http://www.ncbi.nlm.nih.gov/blast) and the Expert Protein Analysis System (http://www.expasy.org/). The protein domains were predicted with the simple modular architecture research tool (SMART) version 4.0 (http://www.smart.embl-heidelberg.de/). The ClustalW Multiple Alignment program (http://www.ebi.ac.uk/clustalw/) was used to create the multiple sequence alignment.

### Real-time PCR analysis of CfCSP mRNA expression

The cDNA mix was diluted to 1∶100 and stored at −80°C for subsequent SYBR Green fluorescent quantitative real-time PCR (RT-PCR). Two CfCSP-specific primers, sense primer P6 and reverse primer P7 (Table 1), were used to amplify the corresponding product of 199 bp. The scallop β-actin, amplified with primers P8 and P9 (Table 1), was chosen as reference gene for internal standardization. DEPC-water for the replacement of cDNA template was used as negative control.

The SYBR Green RT-PCR assay was carried out in an ABI PRISM 7300 Sequence Detection System. The amplifications were conducted in triplicates in a total volume of 25 µL. Dissociation curve analysis of amplification products was performed at the end of each PCR reaction to confirm that only one PCR product was amplified and detected. After the PCR program, data were analyzed with SDS 2.0 software (Applied Biosystems). To maintain consistency, the baseline was set automatically by the software. The comparative average cycle threshold method was used to analyze the mRNA expression level of CfCSP, and the value stood for an n-fold difference relative to the calibrator [44]. All data were given in terms of relative mRNA expressed as mean ± S.E. (N = 5). Differences were considered extremely significant at *P*<0.01 and significant at *P*<0.05.

### Recombinant expression of CfCSP and purification of the fusion protein

The cDNA fragment encoding the mature peptide of CfCSP was amplified with the primers P12 and P13 (Table 1). A *Sac* I site was added to the 5′ end of primer P12 and a *Hind* III site was added to the 5′ end of primer P13 after the stop codon. The PCR fragment was digested with restriction enzymes *Sac* I and *Hind* III (NEB), and ligated into predigested expression vector pET-30a (Novagen). The recombinant plasmid (pET-30a-CfCSP) was transformed into *E. coli* BL21 (DE3)-pLysS (Novagen). The recombinant CfCSP protein was purified with a Ni^2+^ chelating Sepharose column, and the purified protein was refolded as previously described [45]. Then the resultant protein was separated by reducing 15% SDS-polyacrylamide gel electrophoresis (SDS-PAGE), and visualized with Coomassie Bright Blue R250. The concentration of purified rCfCSP was quantified by BCA method [46].

### Nucleic acid binding analysis

Gel retardation analysis with ss/dsDNA substrates was performed as previously described [33]. Totally 150 ng of either single-stranded (M13mp8) or double-stranded (M13mp8 RFI) DNA (NEB) was incubated with the his-tagged rCfCSP proteins in amounts ranging from 0, 20, 100, to 300 pmol. Nucleotide and protein were incubated in 15 µL of binding buffer (10 mM Tris-HCl, pH 7.5) at 0°C for 30 min.

Luciferase (luc) mRNA was *in vitro* transcribed from a sequence-confirmed firefly luc plasmid with a RiboMAX kit (Promega). For *in vitro* transcription, two luc gene specific primers, sense primer P10 and antisense P11 (Table 1) were designed and the phage T7 promoter was added to the 5′ end of primer P10. The DNA template generated by PCR was purified and transcribed with T7 RNA polymerase using the RiboMAX protocol (Promega). The *in vitro* transcribed luc mRNA and the his-tagged rCfCSP protein were incubated in 15 µL of binding buffer (10 mM Tris-HCl, pH 7.5) at 0°C for 30 min. The purified rCfCSP proteins were added to binding reactions in amounts of 0, 20, 60, 120, and 240 pmol. The binding reactions were subjected to agarose gel electrophoresis and visualized by ethidium bromide staining [47].

### Bacterial Complementation

For bacterial complementation studies, the cDNA fragment encoding the mature peptide of CfCSP was amplified with the primers P14 and P15 (Table 1), while the cDNA fragment encoding the *E. coli* CSPA was amplified from the strain ‘BL21-Gold (DE3) pLysS AG’ with the primers P16 and P17 (Table 1). An *Nde* I site was added to the 5′ end of primer P14 and P16, while an *EcoR* I site was added to the 5′ end of primer P15 and P17. The PCR fragments were digested with restriction enzymes *Nde* I and *EcoR* I (NEB), and ligated into predigested expression vectors pINIII. These pINIII constructs (pINIII-CfCSP and pINIII-CSPA) were transformed into *E. coli* BX04 (Δ*cspA*Δ*cspB*Δ*cspE*Δ*cspG*) cells [12]. The *E. coli* stain JM109 with pINIII plasmid was selected as positive control and the *E. coli* stain BX04 with pINIII plasmid was selected as negative control. Overnight cultures of *E. coli* with respective plasmid were streaked on LB-ampiciline plates and grown at either 37 or 17°C.

## Results

### The sequence characters of CfCSP cDNA

**Figure 1:**
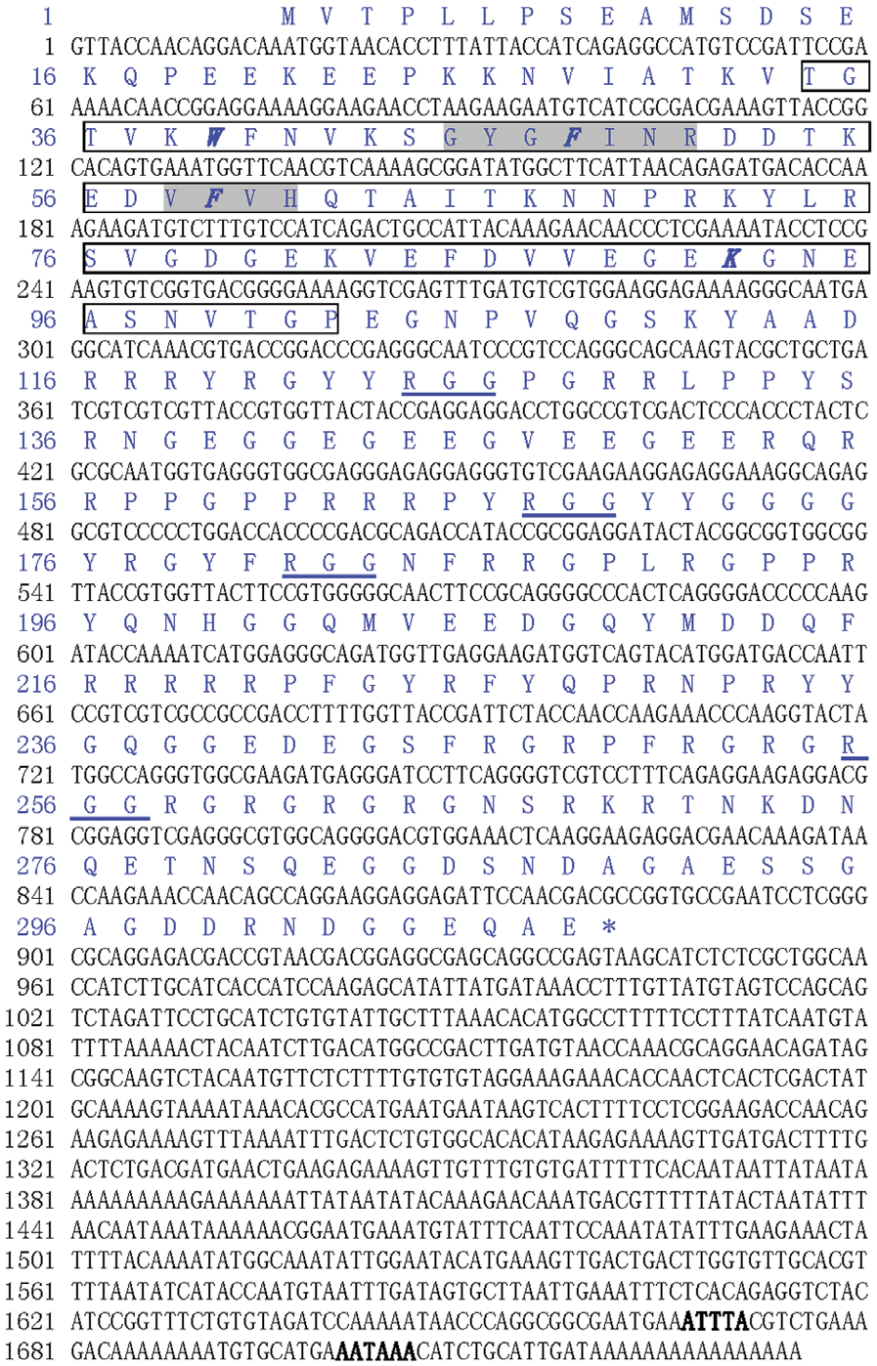
Nucleotide and deduced amino acid sequences of CfCSP. The nucleotides and amino acids are numbered along the left margin. The putative CSD is boxed. The two consensus RNA binding domains are shaded in gray and four DNA binding sites are marked in italic and bold. The RGG repeat motifs are underlined. The classical polyadenylation signals and the A+U destabilizing elements in the 3′ UTR are marked in bold.

A PCR product of 565 bp was amplified from cDNA template by 3′ RACE. By overlapping this segment with EST no. rscag0_004862, a full-length CfCSP cDNA of 1735 bp was obtained (Fig. 1A) and deposited in GenBank under accession JN869460. It included a 5′-untranslated regions (UTRs) of 16 bp, a 3′-UTRs of 792 bp with a classical polyadenylation signal (AATAAA) and one A+U destabilizing elements (ATTTA), and an open reading frame (ORF) of 927 bp encoding a polypeptide of 308 amino acids with a predicted molecular weight of 34.57 kDa and theoretical isoelectric point (pI) of 9.69 (Fig. 1A).

Several distinct functional domains were identified in the deduced amino acid sequence of CfCSP (Fig. 1). A CSD with two consensus RNA-binding motifs (RNP1 and RNP2) and four DNA binding sites (Trp39, Phe48, Phe59 and Lys92) were located at the N terminus that contributed to the binding of nucleic acids (Fig. 1). Subsequent to the CSD, there were four RGG repeats in the C-terminal domains which were important for invertebrate CSDPs to bind nucleic acids (Fig. 1).

### Homologous analysis of CfCSP

**Figure 2:**
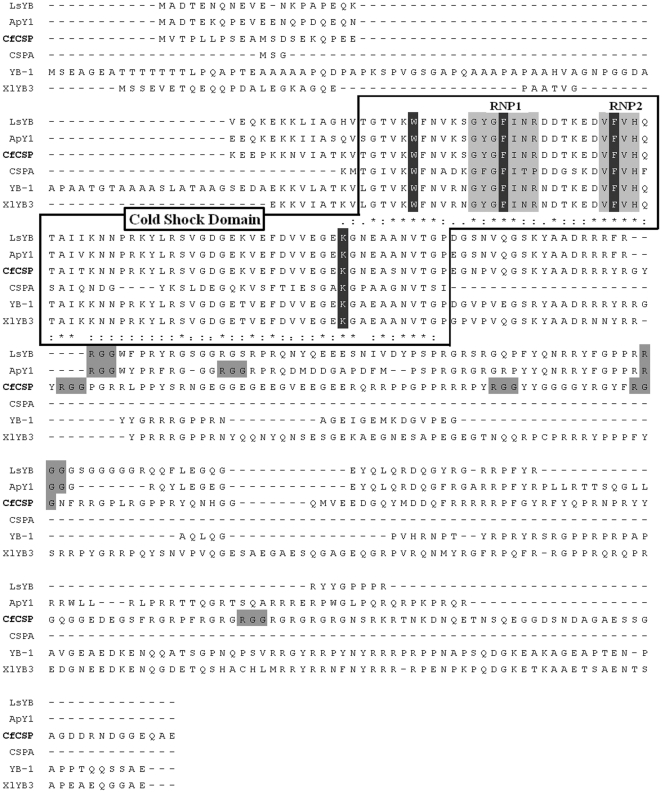
Multiple alignment of the amino acid sequences for CSD containing proteins. Cold shock domains are boxed, consensus RNA binding domains (RNP1 and RNP2) and RGG repeats are shaded in gray and consensus DNA binding sites are shaded in dark. Perfectly matched residues, conserved residues, and less conserved residues are indicated by an asterisk (*), a colon (:), and a period (.), respectively. Accession numbers of the CSD proteins are: CfCSP (JN869460), LsYB (AAT97092), ApY1 (NP_001191560), *E. coli* CSPA (P15277), human YB-1 (P67808) and XlYB3 (CAA42778).

**Figure 3:**
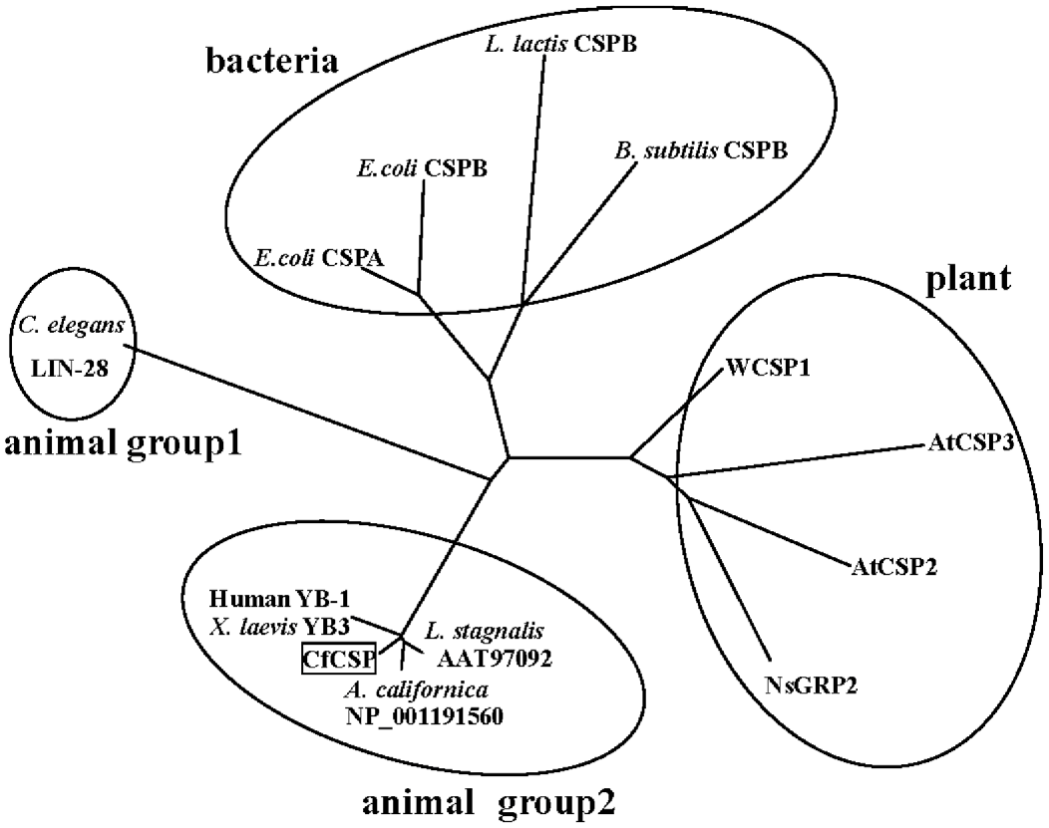
Phylogenetic tree of CSD containing protein sequences from diverse organisms. The tree is constructed by the neighbor-joining (NJ) algorithm and the scale bar corresponds to 0.2 estimated amino-acid substitution spersite. Accession numbers of the CSD containing proteins are: *E. coli* CSPA (P15277), *E. coli* CSPB (P36995), *Lactococcus lactis* CSPB (CAA76695), *Bacillus subtilis* CSPB (P32081), *C. elegans* LIN-28 (AAC47476), Human YB-1 (I39382), CfCSP (JN869460), *Xenopus laevis* YB3 (CAA42778), *Lymnaea stagnalis* YB (AAT97092), *Aplysia californica* Y1 (NP_001191560), WCSP1 (BAB78536), AtCSP3 (NM_127341), AtCSP2 (NP_195580.1) and NsGRP2 (CAA42622).

Sequences of CSDPs from bacteria, invertebrate and vertebrate were downloaded and used for Ident and Sim Analysis (Table 2). The CSD sequence of CfCSP shared significant sequence similarity with that of the CSDPs in invertebrates (96.9% identities to that of *L. stagnalis* and 95.4% to that of *A. californica*) and YB proteins in vertebrates (87.7% identities to YB3 of *X. laevis* and YB-1 of Human). However, the similarity between the CSD sequence of CfCSP and *E. coli* CSPA was relatively low (Percentage identities: 43.1%). The signature sequences of nucleic acid binding motif were identified in CfCSP by multiple sequences alignment (Fig. 2). The amino acid residues among the RNA-binding motif (RNP1 and RNP2) and the DNA binding sites (Trp39, Phe48, Phe59 and Lys92 in CfCSP) were well conserved except that three residues were substituted in the CSPA of *E. coli* (Fig. 2). An unrooted phylogenetic tree with four branches was constructed using neighbor-joining method with 1000 boot-strap test (Fig. 3). The CSPs containing one CSD from bacteria (including CSPA and CSPB of *E. coli*, LlCSPB and BsCSPB) formed one branch. The plant CSDPs containing retroviral-type zinc fingers (including AtCSP2, AtCSP3, WCSP1 and NsGRP2) gathered as another branch. CSDPs from invertebrate (including CfCSP, LsYB and ApY1) and vertebrate (including YB-1 and XlYB3) formed the third one, and the last branch was composed of LIN-28 from *C. elegans* (Fig. 3).

**Table 2:** The percentage identities and similarities of CSD between CfCSP and other CSD containing proteins.

Accession number	Protein name	Organism	I%	S%
AAT97092	Y-box factor-like protein	*Lymnaea stagnalis*	96.9	98.5
NP_001191560	Y-box factor homolog	*Aplysia californica*	95.4	96.9
CAA42778	YB3	*Xenopus laevis*	87.7	90.8
P67808	YB-1	Human	87.7	90.8
P15277	CspA	*Escherichia coli*	43.1	56.9

I%: identity, calculated as the percentage of identical amino acids per position in alignments; S%: similarity, calculated as the percentage of identical plus similar residues. I% and S% were analyzed using the Ident and Sim Analysis provided on http://www.bioinformatics.org/sms/.

### The distribution of CfCSP mRNA in different tissues

**Figure 4:**
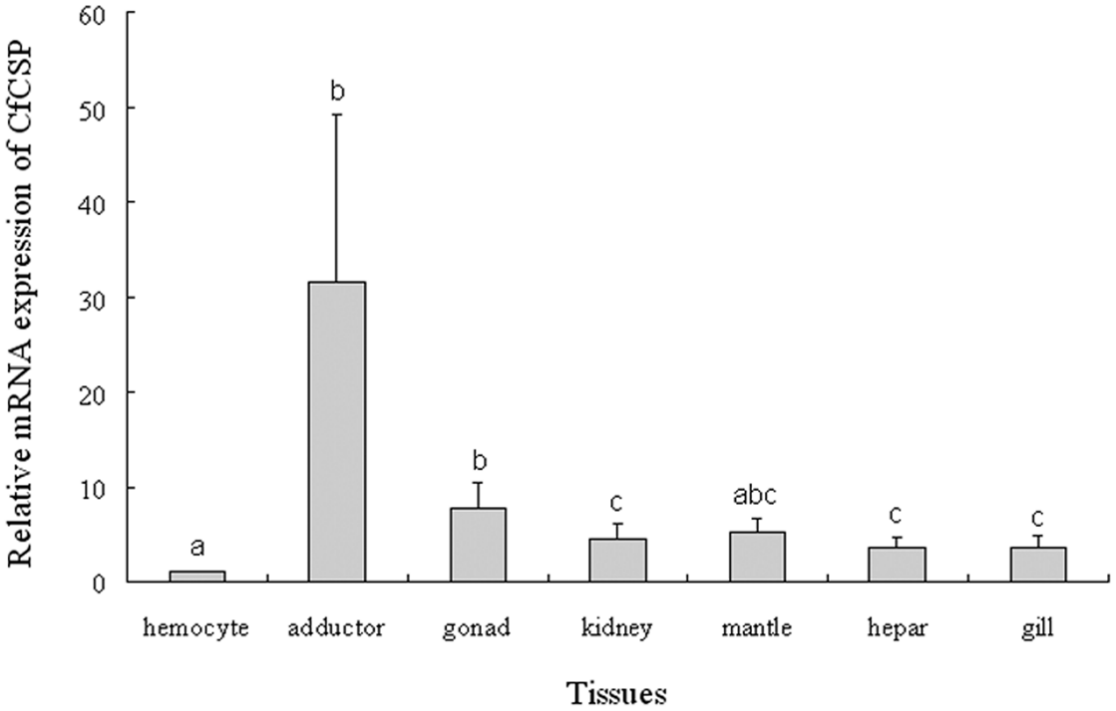
CfCSP mRNA expression in different tissues of *C. farreri* detected by RT-PCR. CfCSP transcripts level in adductor, gonad, kidney, mantle, hepatopancreas and gill were normalized to that of hemocytes. Vertical bars represented the mean ± S.E. (N = 5), and bars with different letters were significantly different (*P*<0.05).

Quantitative real-time RT-PCR was employed to investigate the distribution of CfCSP mRNA in different tissues with β-actin as internal control. For CfCSP and β-actin genes, there was only one peak at the corresponding melting temperature in the dissociation curve analysis, indicating that the PCR was specifically amplified. The CfCSP transcript was ubiquitously detectable in all the tested tissues including gill, hepatopancreas, hemocytes, kidney, mantle, gonad and adductor. The highest CfCSP expression level was found in adductor, which was 31.62-fold of that in hemocytes (*P*<0.01). By contrast, the CfCSP mRNA transcript in gonad, kidney, hepatopancreas and gill was in low level, which was approximately 7.74-, 4.39-, 3.68- and 3.62-fold of that in hemocytes, respectively, and they are significantly higher than that in hemocytes (*P*<0.01). No significant difference was observed between mantle and hemocytes (*P*>0.05) (Fig. 4).

### Temporal expression of CfCSP mRNA in adductor and hemocytes after acute cold shock treatment

**Figure 5:**
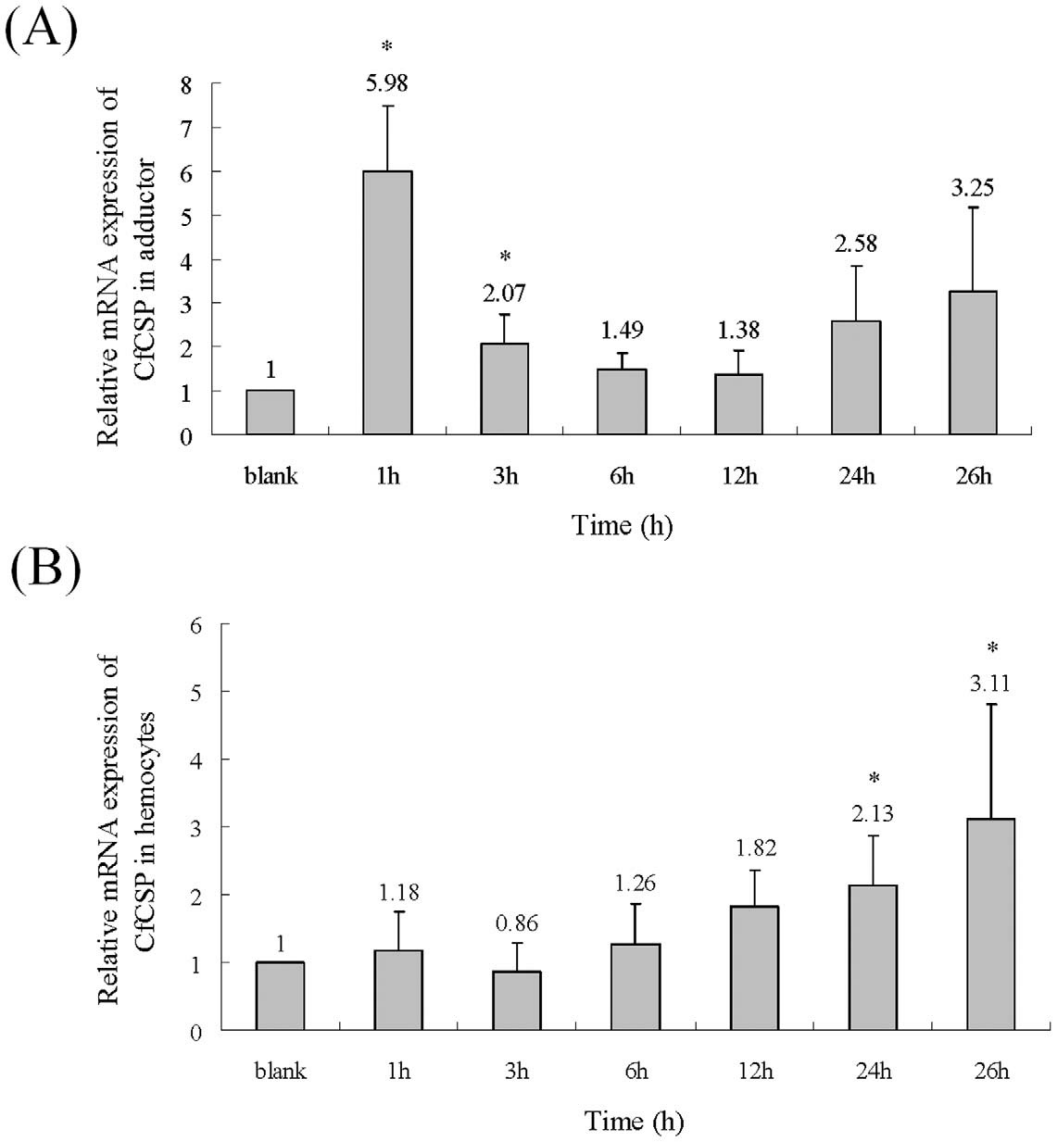
Temporal expression of the CfCSP mRNA after acute cold shock treatment. The mRNA level of CfCSP relative to β-actin in adductor (A) and hemocytes (B) were measured by RT-PCR. Vertical bars represent the mean ± S.E. (N = 5). (*: *P*<0.05).

After acute cold shock treatment, the level of CfCSP transcript in adductor was up-regulated quickly and reached the maximum level of 5.98-fold (*P*<0.05) compared to that in the blank group at 1 h. At 3 h after treatment, the CfCSP mRNA level was still significantly higher (2.07-fold, *P*<0.05) than that of the blank group. As time progressed, the expression level of CfCSP mRNA dropped back to normal level at 6 h (1.49- fold) and 12 h (1.38-fold), and then up-regulated again at 24 h (2.58-fold) and 26 h (3.25-fold), but no significant difference was observed between the treatment and the blank group (*P*>0.05) (Fig. 5A).

After acute cold shock treatment, the mRNA expression level of CfCSP in hemocytes, was up-regulated and reached the maximum level of 3.11-fold (*P*<0.05) at 26 h compared to the blank group. There was no significant difference (*P*>0.05) between the mRNA expression level of CfCSP in treatment and control groups during the first 12 h after treatment (Fig. 5B).

### Recombinant expression of CfCSP in *E. coli*

**Figure 6:**
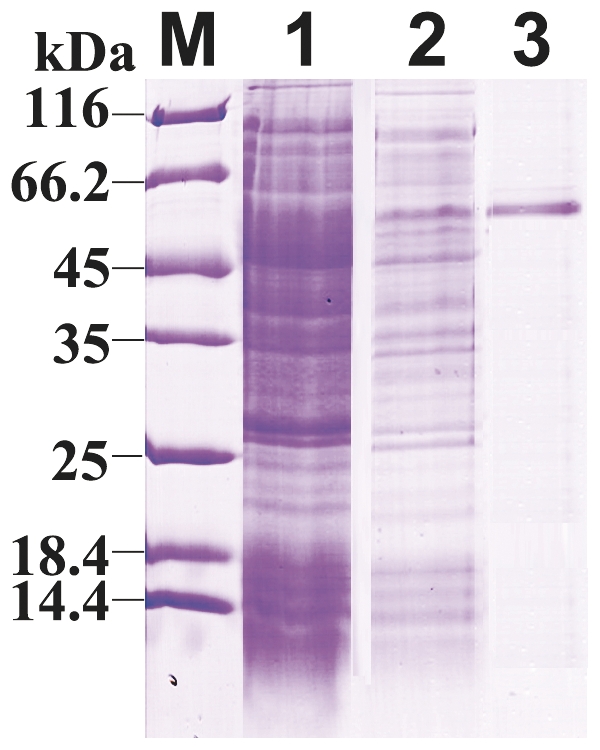
SDS–PAGE analysis of rCfCSP. Lane M: protein molecular standard (kDa); lane 1: negative control for rCfCSP (without induction); lane 2: induced rCfCSP; lane 3: purified rCfCSP.

The plasmid pET-30a-CfCSP was transformed into *E. coli* BL21(DE3)-pLysS. After IPTG induction, the whole cell lysates was analyzed by SDS-PAGE, and a distinct band with molecular weight of ∼53 kDa was revealed, which was consistent with the predicted molecular mass (Fig. 6). The concentration of the purified rCfCSP was 210 µg/mL measured by the BCA assay.

### The nuclear acids binding activities of CfCSP

**Figure 7:**
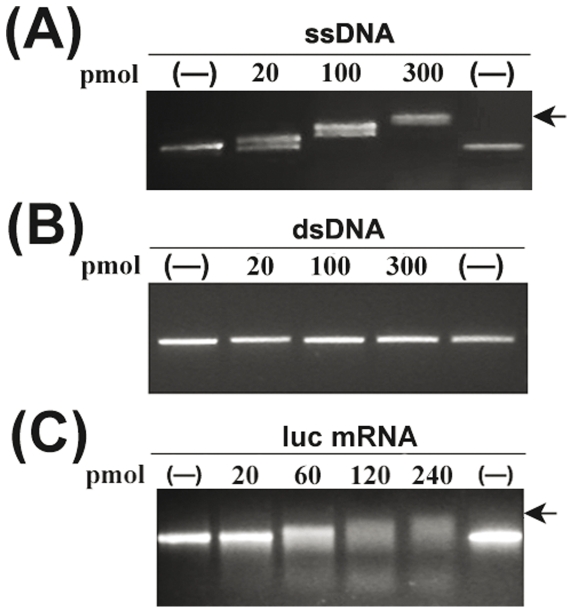
Analysis of nuclear acids binding activity of CfCSP by gel shift assay. The purified rCfCSP proteins were incubated with ssDNA (A), M13mp8 dsDNA RFI DNA (dsDNA) (B) and the *in vitro* transcribed luciferase mRNA (C) to analyze the effect of CfCSP on the formation of nucleotide-protein complexes. A range of CfCSP fusion proteins from 0 to 300 pmol were used for analysis. Arrow shows nucleotide-protein complex.

Gel mobility shift assays utilizing single- or double-stranded phage DNAs as substrates were performed to test the nucleic acid-binding activities of rCfCSP. Shifts of ssDNA bands were detected when 20 pmol rCfCSP was added to the binding reaction (Fig. 7A), and more clear shifts were observed when higher amount (100 pmol or 300 pmol) of rCfCSP was added. However, no shift was detected when dsDNA was incubated with 20 pmol, 100 pmol or 300 pmol rCfCSP proteins (Fig. 7B).

The RNA gel mobility shift assay was performed using *in vitro* transcribed luc mRNA as a substrate. The luc mRNA was incubated with rCfCSP at 0°C in the presence of an RNase inhibitor, and the binding reactions were subjected to 0.8% agarose gel electrophoresis (Fig. 7C). Shifts of luc mRNA bands appeared when the concentration of rCfCSP reached 60 pmol (Fig. 7C, lane 3) and it became more obvious when the amount of rCfCSP increased to 120 or 240 pmol (Fig. 7C, lane 4 and 5).

### CfCSP complements a cold sensitive *E. coli* mutant

**Figure 8:**
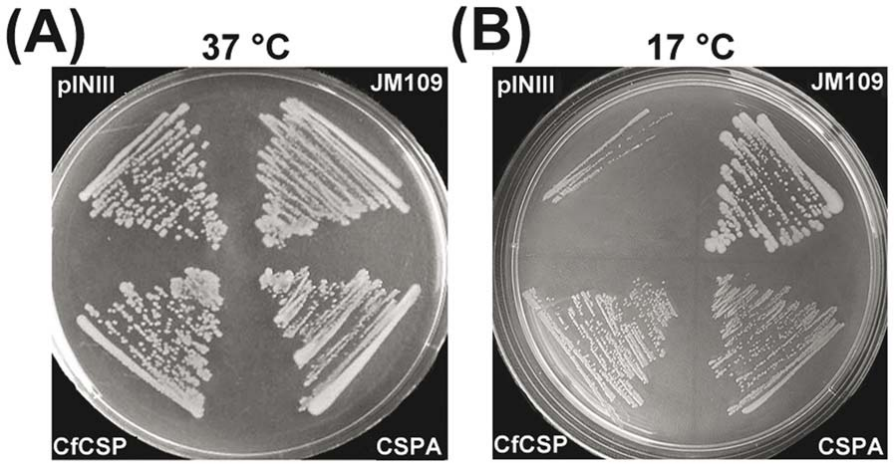
Complementation of cold sensitive growth of BX04 (Δ*cspA*Δ*cspB*Δ*cspE*Δ*cspG*) with CfCSP and *E. coli* CSPA. Overnight cultures of JM109/pINIII, BX04/pINIII, BX04/CfCSP and BX04/CSPA were streaked on LB-ampiciline plates and incubated at 37°C for 12 h (A) or 17°C for 120 h (B).

The pINIII-CSPA/pINIII-CfCSP construction was in BX04 cells confirmed by PCR, and the protein products were also detected by SDS-PAGE (data not shown). After incubation at 37°C for 12 h, the JM109 cells with pINIII and BX04 cells with pINIII, pINIII-CSPA or pINIII-CfCSP all formed large numbers of clones with no difference (Fig. 8A). However, when incubated at 17°C for 120 h, BX04 cells containing the vector pINIII showed growth defect and failed to form colonies, while BX04 cells containing pINIII-CSPA or pINIII-CfCSP showed vigorous growth (Fig. 8B). Though the BX04 cells containing pINIII-CfCSP or pINIII-CSPA also grew evidently at 17°C, the clone was much smaller as compared with the positive clone JM109 with pINIII (Fig. 8B).

## Discussion

CSDPs are nucleic acid-binding proteins involved in various cellular processes, such as cold adaptation [7]. In invertebrates, CSDPs have been identified from several species [35]–[39], but their functions are still not well understood. In the present paper, a CSDP was identified from scallop *C. farreri*. The ORF of CfCSP cDNA was of 927 bp encoding a polypeptide of 308 amino acids. The deduced amino acids of CfCSP consisted of an N-terminal CSD and a C-terminal domain with four RGG repeats. Compared to the bacteria and plant CSDPs, the CSD of the CfCSP displayed higher identity to that of the human YB-1. The presence of CSD in CfCSP suggested that CfCSP might function as a RNA chaperone [14]. In addition to the CSD, there were four additional C-terminal RGG motifs in CfCSP [39]. RGG motif is usually defined as closely spaced Arg-Gly-Gly repeats separated by other amino acids [40], but the four RGG repeats in CfCSP are not closely distributed. The RGG motif is thought to combine with other RNA-binding motifs and functions by increasing the overall RNA affinity of proteins containing additional RNA-binding domain [40]. The difference between the RGG motif from CfCSP and other invertebrate CSDPs might induce some functional changes. These structural information indicated that CfCSP was a new member of invertebrate CSDPs, and it might play a similar role as RNA chaperone at low temperature.

The mRNA expression pattern of CfCSP in different tissue and its temporal expression in adductor and hemocytes after acute cold shock treatment were examined in the present study to explore its possible function. The CfCSP mRNA was constitutively expressed in all the examined scallop tissues, where higher expression level was found in adductor and gonad. However, to our knowledge, the immune or stress related genes are most actively expressed in hepatopancreas or gill, the gene expression pattern of CfCSP gene might have some particular indication for physiological functions. For example, the high expression of cystatin gene in muscle [48], [49] suggested that these proteins might function in muscle cells remodeling. Because the adductor is the main part related to the movement of scallop, the high expression of CfCSP in adductor suggested that it might play similar functions in muscle cells remodeling. After the treatment of an acute cold shock, the CfCSP mRNA expression in both adductor and hemocytes increased significantly (*P*<0.05) within 26 h, indicating that CfCSP probably involved in the acute cold shock response of Zhikong scallops. Considering that the CSDPs from other species always acted as nucleic acid binding proteins [4]–[6], the present results indicated that CfCSP could be effectively induced by acute cold shock and might play an important role in cold adaptation as an RNA chaperone.

The bacterial CSPs could bind RNA and destabilize the secondary structures of RNA to prevent the premature transcription termination under low temperatures [7], [14], [50]. Though many eukaryotic CSDPs displayed DNA/RNA binding activities [4], [19], [23], [25], only RBM3 in Human Cells had been reported in response to cold shock [51]. In CfCSP, a conserved CSD was identified, suggesting that it would exhibit RNA chaperon activity under low temperature. The *in vitro* nucleic acid-binding assay revealed that rCfCSP protein could bind ssDNA and *in vitro* transcribed mRNA, but it could not bind dsDNA. This was different from the Y-box proteins which could bind both ssDNA and dsDNA [4]. It was fascinating that CSPA of *E. coli* acquired dsDNA binding activity by replacing QNDGYK with the Y-box consensus sequence KKNNPRKYLR, suggesting that the length and basicity of the loop region is a determinant of dsDNA binding activity [52]. However, the sequence KKNNPRKYLR in Y-box of human was replaced by TKNNPRKYLR in CfCSP of scallop, and the substitution of the first amino acid from K to T was suspected to lead to the loss of the dsDNA binding activity. Furthermore, the difference of the C-terminal domain with other CSDPs might also result in the loss of the dsDNA binding activity. There were four RGG motifs in C-terminal domain of CfCSP, which was different from other CSDPs in vertebrates and plants. A mutant WCSP1 lacking C-terminal zinc fingers lost dsDNA binding activity, indicating that the C-terminal domain was important for the dsDNA binding.

Similar to the bacterial CSPs, some eukaryotic CSDPs are also suggested to be involved in the process of cold adaptation. In Arabidopsis, GRP7 complemented successfully the cold sensitivity of *E. coli* BX04 mutant and the over-expression of GRP7 increased the cold tolerances of plants [53]. However, GRP4 failed to complement the growth of *E. coli* BX04 mutant during cold stress and over-expression of GRP4 did not increase the cold tolerance of plants [54]. It seemed that there was a common structure-function relationship between bacterial and eukaryotic CSDPs during the process of cold adaptation [33]. In the present study, vigorous growth was observed for BX04 containing pINIII-CfCSP at 17°C, indicating that CfCSP can partially complement the function of *E. coli* CSPs for cold adaptation. Together with the up-regulated expression of CfCSP after acute cold shock, it is reasonable to propose that the abundant transcripts of CfCSP might increase the cold tolerances of scallops. Because of the difference in the process of transcription termination between prokaryotic and eukaryotic systems, it seems that CfCSP can't function as a transcription antiterminator to regulate the cold adaptation in scallops. Meanwhile, wheat WCSP1 has been reported to be involved in the translation process for its ER localization [34], it is suspected that eukaryotic CSDPs might regulate the cold adaptation at translation level.

In conclusion, the present work provided novel information on the roles of mollusk CSDPs in response to acute cold shock. The presence of a conserved CSD and four RGG motifs, the ubiquitous tissue expression and the cold-dependent induction pattern suggested that CfCSP was a primitive member of the invertebrate CSDPs. The nonspecific nuclear acids binding activity and the efficient complementation of the BX04 cold sensitivity suggested that CfCSP offers RNA chaperon activities during the cold adaptation process.
